# Dynamic Assessment of Masonry Towers Based on Terrestrial Radar Interferometer and Accelerometers

**DOI:** 10.3390/s19061319

**Published:** 2019-03-16

**Authors:** Cristina Castagnetti, Elisa Bassoli, Loris Vincenzi, Francesco Mancini

**Affiliations:** Department of Engineering ‘Enzo Ferrari’ (DIEF), University of Modena and Reggio Emilia, 41125 Modena, Italy; elisa.bassoli@unimore.it (E.B.); loris.vincenzi@unimore.it (L.V.); francesco.mancini@unimore.it (F.M.)

**Keywords:** structural health monitoring, ground-based radar interferometry, real aperture radar, accelerometers, bell tower

## Abstract

This paper discusses the performance of a terrestrial radar interferometer for the structural monitoring of ancient masonry towers. High-speed radar interferometry is an innovative and powerful remote sensing technique for the dynamic monitoring of large structures since it is contactless, non-destructive, and able to measure fast displacements on the order of tenths of millimeters. This methodology was tested on a masonry tower of great historical interest, the Saint Prospero bell tower (Northern Italy). To evaluate the quality of the results, data collected from the interferometer were compared and validated with those provided by two types of accelerometer-based measuring systems directly installed on the tower. Dynamic tests were conducted in operational conditions as well as during a bell concert. The first aimed at characterizing the dynamic behavior of the tower, while the second allowed to evaluate the bell swinging effects. Results showed a good agreement among the different measuring systems and demonstrated the potential of the radar interferometry for the dynamic monitoring of structures, with special focus on the need for an accurate design of the geometric aspects of the surveys.

## 1. Introduction

The measurement of structural dynamic responses to ambient and induced excitations contributes to the health monitoring of historic masonry towers and toward the identification of possible damages due to natural or anthropogenic sources and material degradation [1,2]. Traditional health monitoring requires the installation of a number of sensors (accelerometers, strain gauges, and others) on the structures in order to identify global or local structural parameters. Information provided by a reliable survey can be interpreted according to a structural model able to reproduce the structural behavior or as a source of basic parameters in the training of numerical models [3,4]. The accuracy of the damage identification or localization depends on the number and the position of a pre-defined number of sensors and the sensor placement criterion is of great importance to the damage assessment [5,6]. To identify local or small damages, a great number of sensors are usually needed, and some information can only be used for detecting damage in proximity to the installation points. Depending on the structural characteristics, the extensive surveying based on traditional sensors can be expensive and time-consuming and should face limitations related to the preservation of construction integrity or to the accessibility.

In the field of structural health monitoring [7], great benefits have been introduced by terrestrial radar interferometry with a real aperture antenna (also referred to as TInRAR), being such a technique contactless, non-destructive, non-sensitive to dust, and able to detect displacements with an accuracy of tenths of millimeters [8,9,10,11,12,13,14,15,16,17]. Moreover, several authors have introduced benefits in the dynamic surveying of historical buildings and towers from high-speed real aperture radar interferometry under static and dynamic loads, for instance in the procedures devoted to the static and seismic vulnerability analyses [1,18,19,20,21]. Based on sampling rates up to 200 Hz, the TInRAR allows to measure also the fast vibrations of the structure induced by dynamic loadings and to perform the structural dynamic identification in operational conditions. In addition, the use of TInRAR instrumentation can also reveal possible damage to the structures that changes their physical properties and the modal behavior with respect to theoretical responses provided, for instance, by a finite element (FE) model [22]. Some limitations have to be mentioned also for ground-based real-aperture techniques. Basically, they can provide range resolution, and thus, displacements can be detected on the slant direction only. For this reason, geometric aspects of the surveys with TInRAR instrumentation must be carefully designed to detect the structural displacement efficiently.

The available literature has proven in a few case studies the reliability of TInRAR techniques to measure masonry tower oscillations caused by natural excitations or bell ringing, and also by setting the instrumentation 1-km away from the monitored structures [18]. Rarely monitoring data collected from older TInRAR radar sensors has been validated with dynamic data provided by traditional sensors (such as velocimeters or accelerometers) under natural or induced excitations [23,24]. Such a validation procedure needs an accurate design of the experimental tests, a proper definition of the sensor positions in order to allow for a reliable comparison of results, and suitable integration operations when comparing displacements with quantities measured from other types of sensors. Indeed, the integration procedure introduces trends that have to be removed without altering the frequency content of the obtained time-series. The original contribution of this research lies in implementing a rigorous approach that takes into account the abovementioned key issues to provide a reliable validation of a TInRAR-based approach in comparison to a traditional sensors-based approach. The available literature, indeed, often shows TInRAR results and potentialities without a direct comparison to measuring systems based on contact sensors, which present many advantages in terms of quality and reliability of results.

This paper discusses the performances, benefits, and drawbacks of the use of TInRAR technology for structural monitoring of ancient masonry towers. The novelty of the research lies in assessing the usefulness, and consequently the reliability of the provided results, both in integration to traditional sensors and as an alternative in case their use is not feasible. The accuracy of results was evaluated through the comparison of the displacement measured by the radar interferometer with those obtained after a double integration from two types of accelerometers directly installed on the tower. This allowed performing the validation of radar results. The case study was represented by a masonry tower of great historical interest, the tower of Saint Prospero (Reggio Emilia, Northern Italy). Skilled ringers played traditional melodies by moving the tower bells during a famous cultural event held in the historical center of Reggio Emilia. During such an event, the players perceive remarkable displacements that are very difficult to quantify. In this frame, a monitoring of the dynamic behavior of the masonry tower was performed by using an array of accelerometers located at different heights and a TInRAR instrument, manufactured by IDS GeoRadar Srl (IBIS-FS model, a microwave interferometry-based system for remote static and dynamic monitoring). The performances of the TInRAR technology were evaluated both in operational conditions (that is when the tower was subjected to natural excitations) and during a bell concert.

This paper is organized as follows. First, Section 2 presents the tower of Saint Prospero and the dynamic excitation caused by the sound of bells. The measurement systems employed to study the dynamics of the tower, i.e., the terrestrial radar interferometer and the accelerometers, are described in Section 3. Section 4 presents the layout and settings of the measurement systems as well as the techniques adopted to evaluate displacements from measured accelerations and to identify the modal parameters. Time-series obtained from the different measuring systems during the bell concert and modal parameters of the tower are shown and compared in Section 5. Finally, results are critically discussed in Section 6 and conclusions are drawn in Section 7.

## 2. The Case Study

### 2.1. The Tower

**Figure 1:**
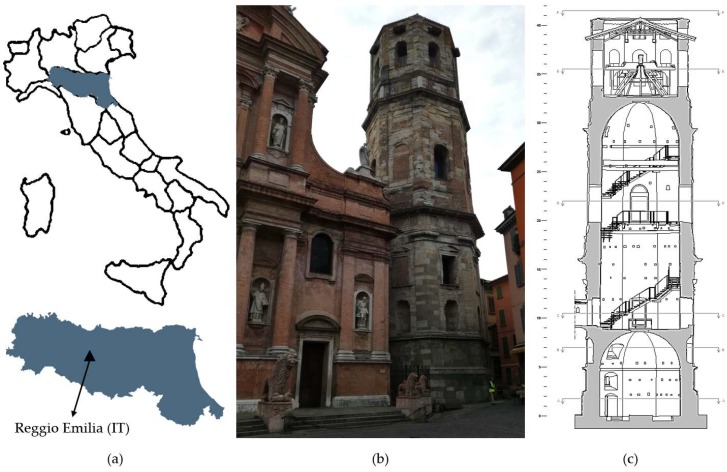
The Tower of Saint Prospero and the nearby Basilica, Reggio Emilia (Northern Italy): (**a**) location map, (**b**) picture of the monuments from the adjacent square, (**c**) internal geometry of the bell tower.

The Basilica of Saint Prospero is a religious building located in Saint Prospero Square, in the heart of the historic center of Reggio Emilia (Emilia Romagna Region, Northern Italy—Figure 1a); it is devoted to the Patron Saint of the city and represents a testimony of the Emilian baroque. Next to the Basilica stands its bell tower, called the Tower of Saint Prospero, which is the test structure of this study. The bell tower is located on the right side of the church facade, has an octagonal plan, and is spread over three orders (Doric, Ionic, and Corinthian, from the bottom to the top, respectively), as shown in Figure 1b. It is possible that the choice of the octagon, smoothing the four corners of a square, is due to the narrowness and to the reduced practicability of the chosen site.

The construction of the tower began in 1536 after a troubled phase of approval for the project and lasted more than 30 years with the succession of different architects and responsible technicians within the construction site. The construction ended in 1571 but remained uncompleted due to funding inconveniences (a fourth floor and a dome would be part of the original project). Moreover, the uncommon choice to put together bricks and covering materials based on living stones caused many difficulties. The execution provided a much thicker brick inner part, with a thickness of about 125–130 cm at the level of Doric, with respect to the thickness of the stone coating, ranging from about 8–10 cm to 20–24 cm depending on the parts. Moreover, the lower part of the brick structure shows that recycled material was used, that is inhomogeneous and probably more elastic, while the external coating is more rigid because of the material and the thinner joints. This presumably causes the concentration of the load, for example in the case of earthquakes, on the relatively thin stone coating with the consequent rapid creation of problems for the stones, which therefore tended to detach and/or break. The internal spatiality is characterized by two octagonal vaulted rooms, with the upper one being much higher than the underlying, and a third room, inserted on the top floor of the tower, constituting the bell cell [25] (Figure 1c).

Since the early decades of the seventeenth century, the tower has suffered significant damages to the external marble and sandstone facades, especially due to atmospheric and telluric events, as well as to the lack of adequate ordinary and extraordinary maintenance before 1900. In 1822, the first fall of a tower stone occurred, and in addition to the immediate closure of the nearby access roads, the sound of the bells was stopped as they were identified as being a further cause of detachment of unsafe boulders. The tower was restored for the first time in 1840, and later in 1977, important and complete restoration works were carried out, which involved the architectural structures and the stone facades. Today, the tower again shows problems of detachment of the sandstone coating, and in order to plan long-lasting and effective interventions, a special committee has been activated for the restoration.

### 2.2. The Bell Ring Forcing

**Figure 2:**
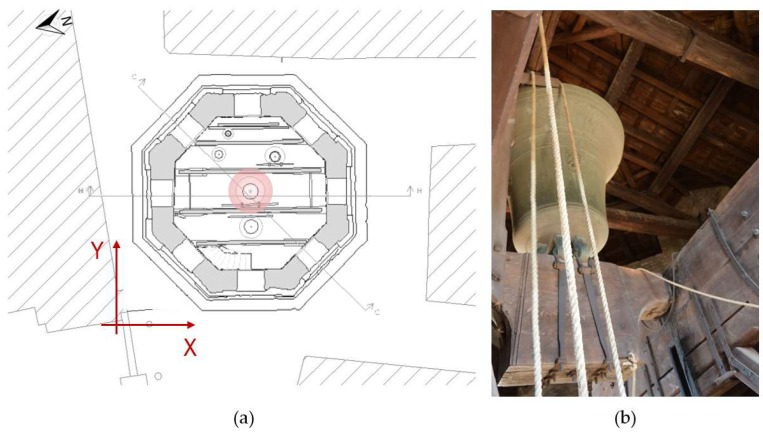
The bell concert of the Saint Prospero Tower: (**a**) the geometry location of the five bronzes of the concert represented in the top view map along with the defined local coordinate system (the red circle highlights the major bell); (**b**) a picture of the upside down position of the major bell just before starting the traditional “suonata distesa”.

The current bell concert dates back to 1796; it is composed of five pieces and was created and masterfully mounted on a wooden castle, the same age as the bells, that is still perfectly preserved today [26]. The five bells are characterized by a weight of about 2.4 tons, 1.2 tons, 0.7 tons, 0.3 tons, and 0.2 tons, respectively from the biggest to the smallest, and a major diameter of 1.5 m, 1.2 m, 1.0 m, 0.8 m, and 0.6 m, respectively (Figure 2a).

One of the peculiarities of this case study lies in the type of forcing used for the dynamic tests: the bells are rung according to the traditional technique, known as “suonata distesa”. This expression is generally used to indicate the bell that oscillates around an almost barycentric axis. During the oscillation, the clapper receives an acceleration due to the relative motion with the bell, and this determines during the ascent semi-oscillation a discrepancy between the decelerations leading to the striking and bell impact. In order to perform this type of sound, it is necessary to have a large number of expert bell ringers (about a dozen), as this is a rather complex technique. In fact, while the bell ringers pull the ropes in order to move the major bell, one more ringer performs articulated melodies with the minor bronzes. Meanwhile, the other bell ringers standing on the wooden frame, from a raised position that coincides more or less with the top of the oscillating movement of the bell, guarantee the regularity of the oscillation time that dictates the metric of the execution. In this experimentation, the major bell is brought into a vertical position with a series of tied swinging oscillations (the bell oscillates 360 degrees, each time in opposite directions). Once it is upside down, it is oscillated repeatedly, thanks to the expertise of the bell ringers that impose different forces depending on the melody to ring (Figure 2b).

## 3. Sensors

### 3.1. The Real Aperture Radar (RAR) Terrestrial Interferometer

The terrestrial radar interferometer used in this study (IBIS-FS model, IDS GeoRadar Srl manufacturing) is able to detect differential displacements by comparing the back-reflected phase information of the radar signal collected at different times with respect to the transmitted one [1,27]. Displacements along the line of sight (LoS) of the order of 0.1 mm can be detected by the radar interferometer at a frequency rate up to 200 Hz. Technical characteristics are shown in Table 1 (for the present experimentation, two antennas operating in the Ku band were mounted, IBIS-ANT3 models) [28]. The simultaneous, 1-dimensional, multi-point measurement was performed by setting-up the instrument at a suitable distance from the monitored structure. The IBIS-FS ability to resolve and detect displacements of different targets in the range direction depends on the radar wave properties.

**Figure 3:**
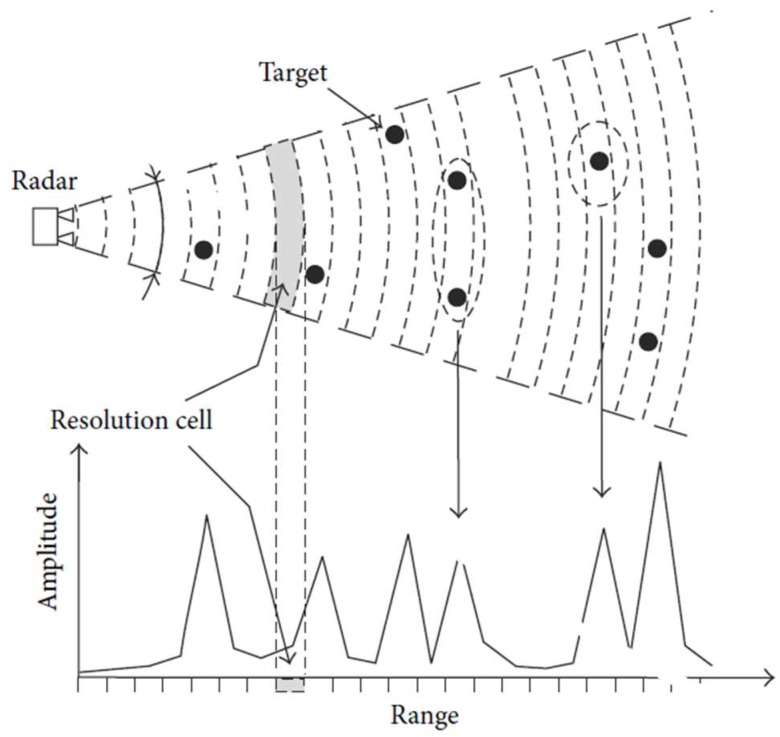
Geometry of an interferometric radar survey from a fixed position and the range resolution principle. Amplitudes of received signals are referred to targets within a resolution cell [27].

In particular, the IBIS-FS uses a linear frequency modulated continuous wave (LFMCW) technique. It guarantees a range resolution of 0.5 m, regardless of the sensor-to-target distance. In Figure 3, the basic principle of the 1D terrestrial radar survey is depicted.

Targets located within a resolution cell from the radar sensor are represented by a single response with no chance to distinguish them whenever a rotation of the radar head is not applied (as in the present investigation). Thus, the azimuth resolution is not available. The displacement along preferred directions can be easily obtained by the knowledge of the acquisition geometry. The portable sensor used operated at 17.1–17.3 GHz, capable of being mounted on a steady tripod and powered by a battery pack. Such characteristics guarantee an easy and fast way to install and disassemble and make the IBIS-FS sensor suitable for a wide range of static and dynamic monitoring with reduced logistic constraints [29].

### 3.2. Accelerometers

Two types of accelerometer-based acquisition systems were adopted to measure the structural response, one built on micro electro-mechanical systems (MEMS) technology and the other on piezoelectric accelerometers. The first was the SHM602 system (Teleco SpA manufacturing), composed of a control and storage unit and six digital bus-connected sensing units. The sensor bus connection assures a high degree of reliability and prevention against electromagnetic interferences. Each sensing unit can record the accelerations along two orthogonal axes and the temperature while the sampling frequency can be selected by the user in the range 20–80 Hz. Thanks to local digital filtering techniques and oversampling rates implemented, these units relying on MEMS sensors can exhibit a noise floor of about 0.3–0.5 mg (where *g* denotes the gravity acceleration) [30,31]. The main features of the SHM602 system are the transmission of data in digital form and the possibility of performing some system analyses directly on-board of the sensors, transmitting the processed synthetic data to the main computer. The main drawback is the limited frequency range, not suitable for stiff structures. The second dynamic acquisition system was composed of 12 uniaxial piezoelectric PCB/393B12 accelerometers with a dynamic range of ±0.5 g, a bandwidth ranging from 0.15 to 1000 Hz, and a resolution of 8 μg. The accelerometers were connected to a National Instruments acquisition system for data storage and system management. The high signal-to-noise ratio of the piezoelectric accelerometers allows a clear acquisition of the structural response in operational conditions even when the wind or traffic excitation is low. On the other hand, the analogic data transmission is very sensitive to external disturbance.

## 4. Data Collection and Processing

The experimental tests conducted on the tower of Saint Prospero aimed at both characterizing the dynamic behaviour of the tower and evaluating the bell swinging effects. The dynamic behaviour of the structure, i.e., its modal parameters, was identified thanks to ambient vibration tests, where the structural responses in operational conditions was recorded. Moreover, the structural response was measured during a bell concert to evaluate and compare maximum displacements in the case of higher levels of excitation.

### 4.1. Monitoring with TInRAR

**Figure 4:**
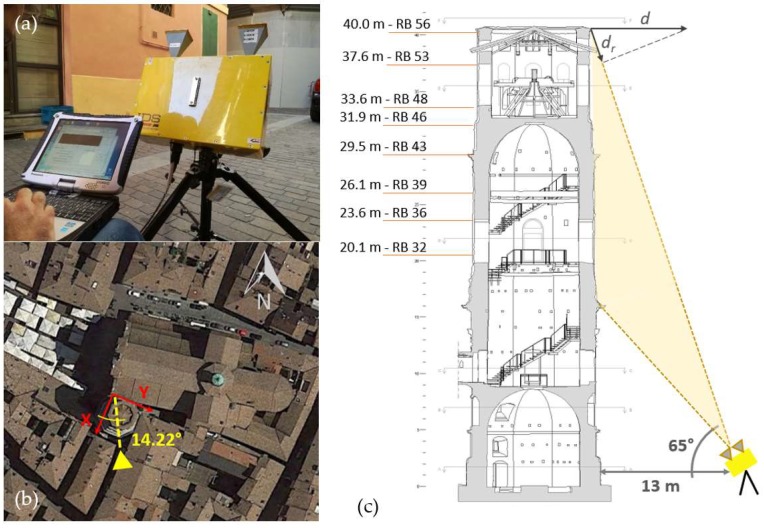
Design of the dynamic monitoring test with the terrestrial radar interferometry with a real aperture antenna (TInRAR) technology: (**a**) picture of the IBIS-FS radar interferometer, manufactured by IDS GeoRadar Srl, and the setup during data collection; (**b**) radar positioning with knowledge of the LoS (line of sight) misalignment with respect to the defined coordinate system; (**c**) geometry setup of the radar with respect to the tower with identifications (location and naming) of the range bins (RBs) selected for analyzing displacement time-series. The principle for computing the radar displacement is also represented in the picture, being *d* the real displacement and *d_r_* the radial displacement (size is exaggerated on purpose).

The dynamic monitoring by means of TInRAR technology needs a careful design. First of all, a local reference system was defined in order to understand and facilitate the interpretation of the tower movements due to the bell ring forcing. A convenient orthogonal Cartesian system was defined taking into account the symmetry of the tower and the position of the major bell. The *x*-axis was parallel to the tower octagon side facing the square, indicatively the west side, and consequently it was approximately oriented along the north–south direction. The *y*-axis was therefore arranged along the west–east direction, parallel to the line joining the centers of the three major bells (Figure 2a and Figure 4b). Based on the defined reference system and considering both the bell positions and the type of forcing, the following basic hypothesis was assumed about the direction of the expected movements: the prevailing motion occurs in the north–south direction, then along the *x*-axis, and no significant component of vibration takes place along the *y*-direction.

According to this hypothesis, the monitoring with TInRAR technology was designed by recalling that the radar instrument is able to detect the components along the LoS of actual displacements, and thus, the instrument should be placed as much as possible orthogonal to the *x*-axis so to make the LoS coincident with the main direction of expected displacements. Due to obstruction of buildings, the instrument was placed in a slightly misaligned position with respect to the *x*-direction that amounted to 14.22 degrees towards east compared to the *x*-axis (see Figure 4b). In cases of more complex movements, that are not characterized by a strong directionality or where a priori reliable hypothesis is not easily definable, it would be necessary to provide further measurement in the orthogonal direction with respect to the first sensor location.

The radar was setup on a tripod in the position defined according to the stated criteria, 13 m from the tower and the antenna was oriented to have an inclination angle of about 65 degrees (Figure 4a,c). As mentioned, such a view angle was necessary to avoid ambiguities among the backscattered radar responses coming from points located at different elevations of the tower. When monitoring high-rise structures with radar technology, indeed, there is a mutual dependence between the number of points that can be observed on the structure at a defined resolution and the ratio of the instrument–structure distance to the height of the structure: the closer the radar is to the structure, the more points are observed [29]. The spatial resolution used in the present test was 0.75 m. The acquisition frequency during the whole test was set to 100 Hz; the overall test lasted about 1 h, with a first step of about 10 min sensing the structure under natural conditions, and a following step under dynamic conditions where the bells rung during three events, each one characterized by a different melody.

Raw radar observations were processed by means of the IBIS Data Viewer software, version 03.05. The parameters of the geometric survey were introduced in the software before starting the data processing. Defining the geometry allowed conversion of the radial displacement values *d_r_*, i.e., along the axis of the radar beam, into real displacements *d* (horizontal in case of towers—see Figure 4c). Once the data processing was performed, the next step was to analyze the range profile in order to select appropriate points located on the tower. The range profile is shown as a graph and is composed of points of the width equal to the range resolution. These points are called range bins (RBs) and are numbered sequentially, starting from the position of the radar unit [29]. Being the measurement geometry properly defined in the processing options, the location of the selected points on the structure was inferred. Specific structure points were selected within the range profile (i.e., in relation to the location of accelerometers) taking also into account the quality of signal power. Any selected point was included in a list that displays additional information such as range bin number (that is used as identification of the points in the following), the distance from the radar, and the distance from the beginning of the structure (i.e., for the tower this means the elevation of the point). The range bins selected for the present study are displayed in Figure 4c. For each range bin, the time-series of horizontal displacements (i.e., as computed starting from the radial displacements) were provided in order to perform analyses and comparison with the results of the accelerometer-based systems.

### 4.2. Monitoring with Accelerometers

**Figure 5:**
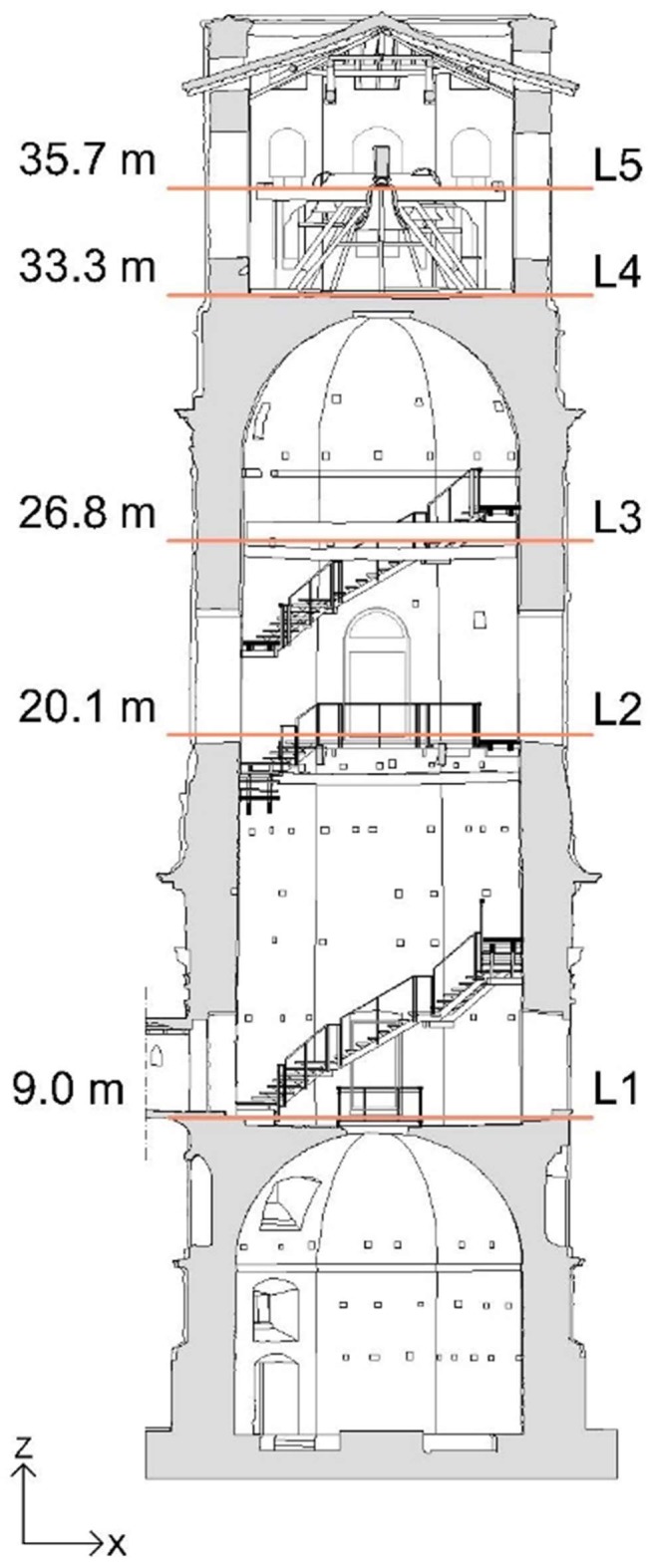
The instrumented levels (L1–L5).

**Figure 6:**
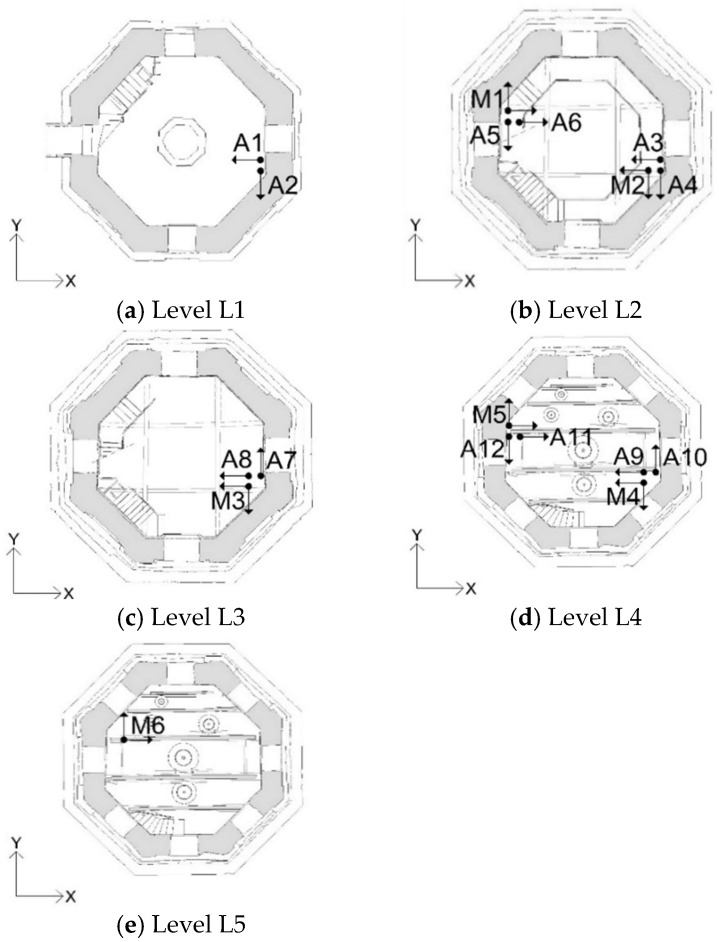
Layout of the piezoelectric (A1–A12) and micro electro-mechanical systems (MEMS)-based (M1–M6) accelerometers.

**Figure 7:**
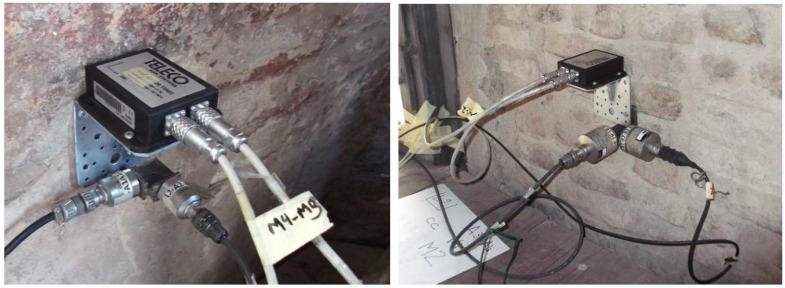
Typical installation of MEMS and piezoelectric sensors.

The dynamic responses of the tower were measured simultaneously in seven points belonging to five levels (L1–L5 in Figure 5). In each measuring point (except for those at levels L1 and L5), one biaxial MEMS sensor and two uniaxial piezoelectric accelerometers were placed in order to compare the accelerations acquired from the two measuring systems. A total of 12 piezoelectric accelerometers (A1–A12 in Figure 6) and six MEMS accelerometers (M1–M6 in Figure 6) were employed. The level L1 was monitored using only piezoelectric accelerometers (Figure 6a). At the level L5, the accelerometer M6 was placed on a beam of the wooden frame that supports the bells (Figure 6e). Except for this last accelerometer, the others were installed on the inner walls by means of metal plates and screws, as shown in Figure 7. The sampling frequency was set to 200 Hz and 80 Hz for the piezoelectric and the MEMS accelerometers, respectively.

To compare the results with those given by the terrestrial radar interferometer, the displacement of the tower was evaluated from the recorded acceleration through a double numerical integration performed adopting the Simpson’s rule. This procedure introduces unavoidable errors due to noise amplifications, as discussed in Reference [32]. To limit the effect of these errors, a three-step procedure was carried out. In the first step, the double integration of the measured accelerations with the Simpson’s rule was performed. Accelerations were directly used without any signal pre-processing in order to avoid the removal of any important frequency content. In the second step, a de-trending procedure was applied based on the so-called “empirical mode decomposition” (EMD) [33]. The EMD method is an adaptive method that expresses the original signal as the sum of intrinsic mode functions (IMF) *h_i_*(*t*) and the residual *r*(*t*):

$$x\left(t\right)=\sum_{i=1}^{n}h_{i}\left(t\right)+r\left(t\right)$$

where the *i*-th IMF *h_i_*(*t*) is signal dependent and is extrapolated sequentially from the signal by the sifting algorithm described in References [33,34]. Each IMF represents a narrow-banded oscillation embedded in the data, while the residual represents the signal trend.

In contrast to conventional decomposition methods, such as Wavelets [35] that perform the analysis by projecting the signal into a number of predefined basis vectors, the EMD method is fully data-driven, since the decomposition is adaptive to the signal itself. For this reason, it is also suitable for non-stationary and non-linear data. Since the residual represents the signal trend, the processed signal was computed subtracting the residual to the originally acquired acceleration.

Finally, in the third step, a high-pass standard filter was implemented in order to remove the low-frequency content introduced by the integration procedure (de-noising step).

### 4.3. Modal Parameter Estimate

The dynamic properties of the tower were estimated from the acceleration recorded in operational conditions. Due to the high signal-to-noise ratio of the piezoelectric accelerometers, the dynamic identification was performed only with reference to the acceleration measured from the piezoelectric system. The modal identification was carried out adopting the enhanced frequency domain decomposition (EFDD) method [36,37]. The method relies on a singular value decomposition (SVD) of the power spectral density (PSD) matrix of the acquired accelerations. The *j*-th natural frequency was identified from the peak of the PSD graph, while the singular vector of the PSD matrix and the corresponding singular value represent, respectively, the *j*-th mode shape and the amplification factor. Finally, the damping ratio was estimated through the logarithmic decrement. The reader is referred to References [36,37] for all the details about the method.

## 5. Results

### 5.1. Comparison among Time-Series during the Bell Ring Forcing

**Figure 8:**
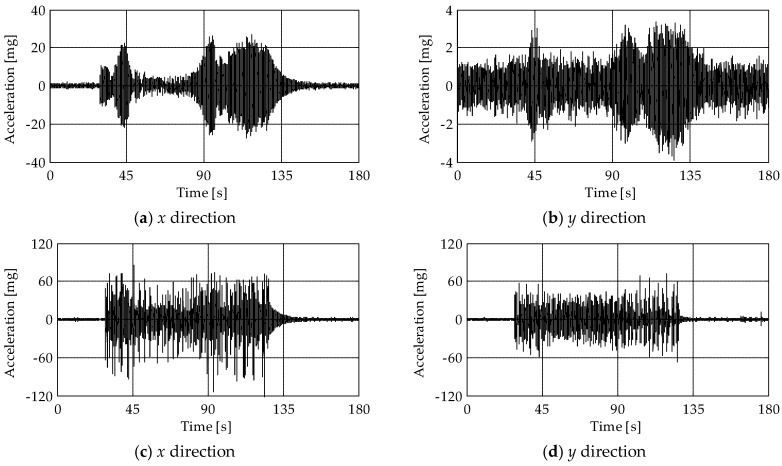
Acceleration measured from the sensors (**a**,**b**) M4 and (**c**,**d**) M6 in the *x-* (left column) and *y-* (right column) directions during the first event of the bell concert.

**Figure 9:**
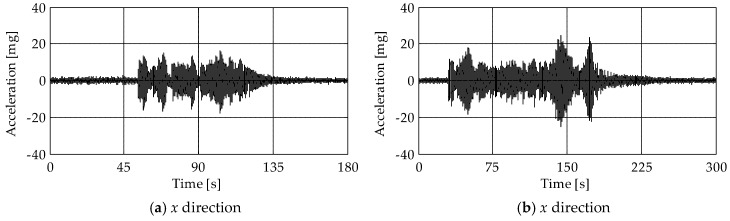
Acceleration measured from the sensor M4 in the *x*-direction during the (**a**) second and (**b**) third event of the bell concert.

To evaluate the structural response of the tower to the bell swinging, experimental tests were conducted during a bell concert. The bell concert can be divided into three main events. In the first event (Figure 8), the major bell was brought into a vertical position with a series of tied swinging oscillations, while in the last two events (Figure 9), two different melodies were performed. Figure 8 shows the acceleration recorded by the MEMS sensors M4 and M6 (placed at levels L4 and L5, see Figure 5) during the first event in the *x*- and *y*-directions. Considering the sensor M4 (Figure 8a,b), maximum accelerations of about 25 mg were observed in the *x*-direction, i.e., the direction of the oscillation of the bells, while in the *y*-direction accelerations were one order of magnitude lower. On the contrary, accelerations of the wooden frame supporting the bells, measured by the sensor M6 (Figure 8c,d), were between ±60 mg in both the *x*- and *y*-directions, with peaks up to 120 mg in the *x*-direction. Finally, accelerations measured by the sensor M4 in the *x*-direction during the second and third event (Figure 9a,b) were close to those of the first event.

**Figure 10:**
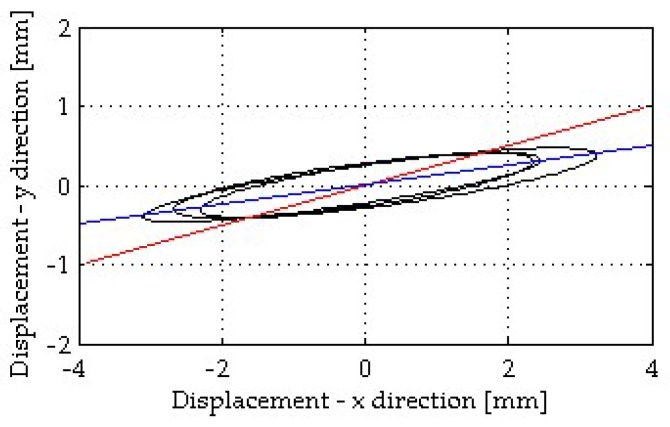
Displacement in the *x*- and *y*-directions of the level L4 (black line): sensors A11 (*x*-direction) and A12 (*y*-direction). Blue line: direction of maximum displacement (7.05° from the *x*-direction); red line: measuring direction of the TInRAR (14.22° from the *x*-direction).

Displacements of the accelerometer-based measuring systems were calculated from the recorded accelerations through a double numerical integration, as reported in Section 4.2. Figure 10 plots the displacement of the level L4 in the *x*- and *y*-directions during a few seconds of high excitation due to the bells. As expected, the tower oscillated mainly in the *x*-direction although non-negligible oscillations were observed also in the *y*-direction. Indeed, the direction of maximum displacement of the tower was tilted 7.05° from the *x*-direction. Figure 10 also shows the misalignment of the measuring direction of the terrestrial radar interferometer (14.22°) with respect to the *x*-direction. To compare the results of the accelerometers (that measure in the *x*- and *y*-directions) with those obtained from the TInRAR, accelerations were projected on the same measuring directions of the TInRAR.

**Figure 11:**
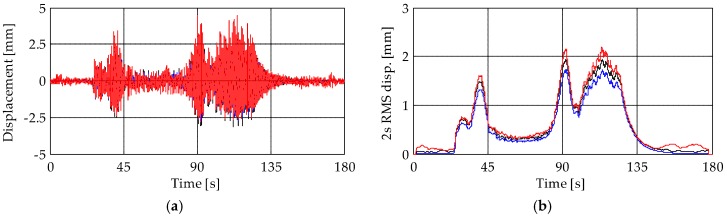
(**a**) Displacements and (**b**) root-mean-square (RMS) displacement traces of level L3. Black lines: MEMS accelerometer M3; blue lines: piezoelectric accelerometer A8; red lines: TInRAR, range bin 39.

**Figure 12:**
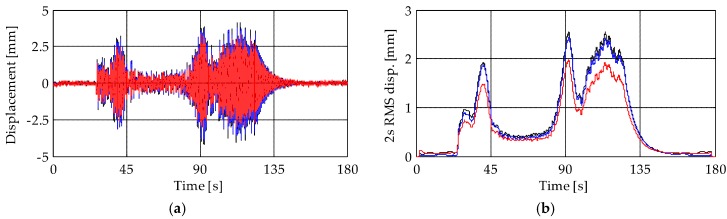
(**a**) Displacements and (**b**) RMS displacement traces of level L4. Black lines: MEMS accelerometer M4; blue lines: piezoelectric accelerometer A9; red lines: TInRAR, range bin 48.

**Figure 13:**
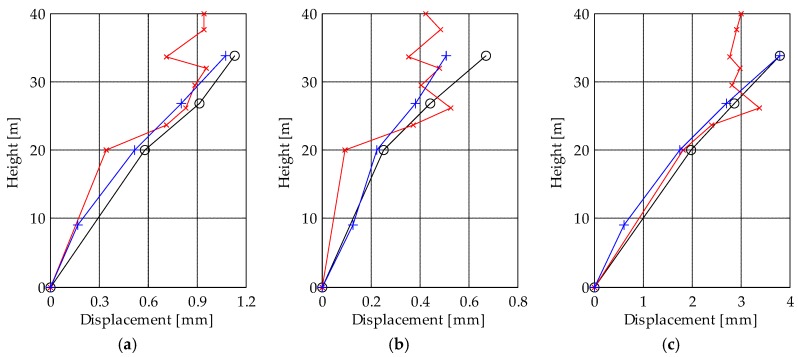
Displacement along the height of the tower at three time instants: (**a**) t = 30.8 s, (**b**) t = 63.5 s, (**c**) t = 92.9 s. Black lines: MEMS accelerometers; blue lines: piezoelectric accelerometers; red lines: TInRAR.

**Figure 14:**
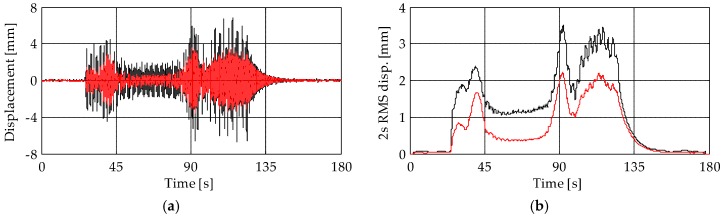
(**a**) Displacements and (**b**) RMS displacement traces of level L5. Black lines: MEMS accelerometer M6; red lines: TInRAR, range bin 53. Note that in this case, the accelerometer measured the displacement of the wooden frame supporting the bells and not the displacement of the tower itself.

**Figure 15:**
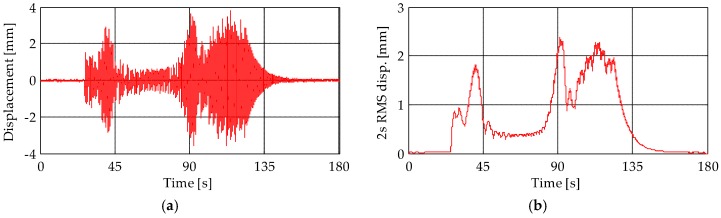
(**a**) Displacements and (**b**) RMS displacement trace measured by the TInRAR (range bin 56) at the height of 40.00 m.

**Figure 16:**
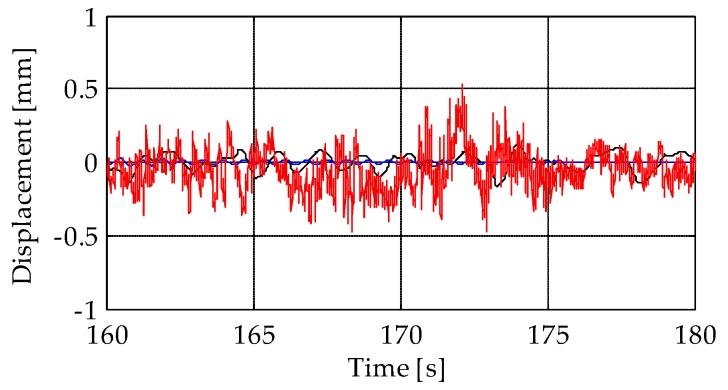
Detail of the time-series of level L3 after the bell sound (free vibrations). Black lines: MEMS accelerometer M3; blue lines: piezoelectric accelerometer A8; red lines: terrestrial radar interferometer.

**Figure 17:**
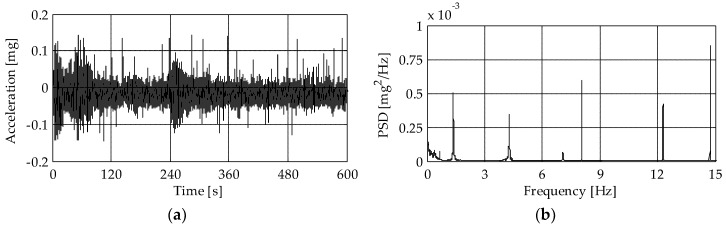
(**a**) Typical acceleration time-series recorded at level L4 in operational condition and (**b**) corresponding PSD function.

Results obtained from the accelerometers (MEMS and piezoelectric) and the terrestrial radar interferometer are compared in Figure 11, Figure 12, Figure 13, Figure 14, Figure 15, Figure 16 and Figure 17. They are presented in terms of both displacement and maximum root-mean-square (RMS) values of the displacement with an averaging period of 2 s. For the sake of simplicity, results presented in the following refer to the first event of the bell concert. However, the analysis of the other two events leads to similar results and allows drawing the same conclusions. 

Figure 11 and Figure 12 present the displacements of levels L3 and L4, respectively. A close match between the results of the two accelerometer-based systems can be observed. In addition, results show that, depending on the analyzed level, the TInRAR can slightly overestimate or underestimate the displacements measured by the accelerometers. However, differences of peaks are, on average, of the order of 0.20 mm (over about 4 mm of peak displacement that corresponds to the 5%) with a maximum difference of 1.28 mm (about 32%), stating quite good correspondence among results. On the contrary, differences of peaks between piezoelectric and MEMS accelerometers are lower, with a mean value slightly lower than 0.10 mm (about 2%) and a maximum difference of 0.52 mm (about 14%). These differences are, in general, appreciable but not so large to compromise the reliability of results.

The main drawback related to these differences is evident when the complete deformed shape of the tower is computed for a selected time instant. For example, Figure 13 shows the displacement of the measuring points along the height of the tower at three different time instants. As mentioned before, the displacements of the two accelerometer-based measuring systems are consistent with each other while the TInRAR underestimates or overestimates the results depending on the considered level as well as the time instant. Indeed, the deformed shape obtained from the TInRAR presents abnormalities along the height of the tower, which are not present in those obtained from the accelerometers.

The displacements of the level L5 obtained from the MEMS accelerometer M6 and TInRAR are compared in Figure 14. In this case, the significant discrepancy between results is because the MEMS accelerometer was placed on the wooden frame supporting the bells instead of on the tower, while the TInRAR measured the displacement of the tower itself. Indeed, the wooden frame is more flexible than the tower and suffers from higher displacements due to the bells. Moreover, Figure 15 shows the displacement measured from the TInRAR at the top of the tower (40.00 m). At that level, the corresponding structural response measured from the accelerometers was not available because of accessibility problems. In general, results show that the maximum displacement caused by the bell swinging goes up to about 5 mm. 

By analysing a few seconds of free vibrations after the bell concert (Figure 16), it can be observed that displacements measured from the TInRAR were characterized by higher noise levels than those obtained from the accelerometers.

Finally, it is worth stressing that the displacements measured by the TInRAR were slightly ahead of time with respect to those obtained from the accelerometer-based systems. More precisely, the TInRAR measured 1.5 milliseconds less each second with an error in time of 0.15%. This caused a time shift among the measured signals that can be observed after about 100 s of measurements. Being that the MEMS and piezoelectric accelerometers are based on two different and independent measuring systems, it is reasonable to assume that the TInRAR lacks precision and not vice versa. However, it should be emphasized that this discrepancy does not affect the results of the analyses aimed at evaluating the structural displacements and characterizing the dynamic behavior of structures. Indeed, in the frequency domain, this time shift caused an error of about 0.15% when estimating the frequency of a peak. This error is of the same order of magnitude of the uncertainties in the natural frequency estimate typical of an efficient identification algorithm.

### 5.2. Comparison of the Frequency Content

**Figure 18:**
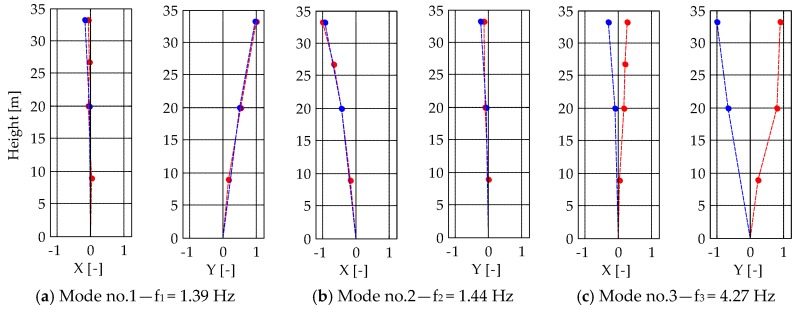
Mode shapes of the identified (**a**,**b**) bending and (**c**) torsional modes. Red: modal displacement of the measurement points in the right corner of the cross-section. Blue: modal displacement of the measurement points in the left corner of the cross-section.

Figure 17a shows a typical acceleration time-series recorded by the piezoelectric accelerometers at the upper instrumented level (L4) while the corresponding PSD function is presented in Figure 17b. The measured acceleration ranged between ±0.15 mg, stating the low level of excitation during the ambient vibration test. Thanks to the identification procedure reported in Section 4.3, six natural modes were clearly identified. To give an example, Figure 18 presents the natural frequencies and corresponding mode shapes of the first three modes. The first two modes are dominant bending and involve flexure in the *y-* (Figure 18a) and *x-*directions (Figure 18b), while the third mode (Figure 18c) mainly involve torsion of the tower. The damping ratios of the identified modes ranged between 0.8% and 1.8%. However, due to the non-linear effects, the damping ratio depends on the forcing amplitude. Consequently, higher values of damping ratios would be obtained if they were identified from the free decay oscillations after the bell ringing.

**Figure 19:**
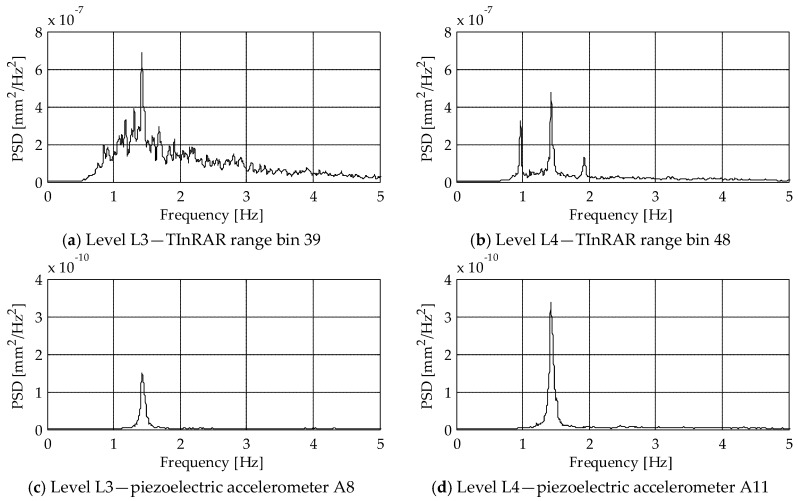

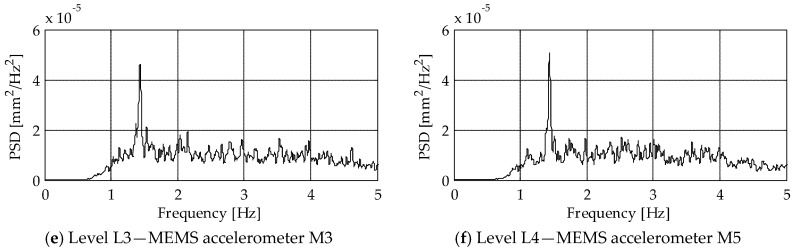
PSD calculated at level L3 (left column) and L4 (right column) from the radar (first row), the piezoelectric accelerometers (second row) and the MEMS accelerometers (third row).

For a preliminary assessment of the performance of the TInRAR in the case of ambient vibrations, Figure 19 reports the PSD functions calculated from the displacements of the three measuring systems in operational conditions. The piezoelectric accelerometers allowed for a clear identification of the peak at 1.44 Hz (Mode n.2 in Figure 18) at both levels L3 (Figure 19c) and L4 (Figure 19d), proving the high precision of these accelerometers even in the case of low excitation. As regards the MEMS accelerometers, the same peak can be recognized at both levels (Figure 19e,f), although the PSD functions are characterized by higher noise. The same goes for the TinRAR, which allowed for a pretty clear identification of the peak at both levels (Figure 19a,b), but presented a PSD for the level L3 characterized by high noise.

It is worth noting that from the TInRAR, only ten minutes of measurements in ambient conditions were available. Hence, for a proper comparison, also the PSD functions of the accelerometer-based systems were calculated with reference to the same time interval. However, the analysis of different and longer time-series allows (especially with the piezoelectric accelerometers) obtaining PSD functions where several peaks can be observed, as the one reported in Figure 17b.

## 6. Discussion

The TInRAR technology proved to be in good agreement with the traditional sensors that are conventionally used to assess the dynamics of a structure. During the bell concert, the tower experienced vibrations that were recorded by all sensors, i.e., the accelerometers installed inside the tower and the radar from the remote location. The analysis of the maximum displacement along the height of the tower (Figure 13) shows that the two kinds of accelerometers were very consistent while the radar sometimes slightly differed. The comparison among displacement time-series shows differences on average of 5%, with peaks of 32%. These differences are not very relevant for analyses aimed at evaluating the frequency content of the signal or the maximum displacements. On the other hand, they can cause the noisy deformed shape of Figure 13 that can limit the use of the TInRAR only for flexible structures or for detecting slow movements. In this case, a careful strategy when using radar observations only would be to analyze multiple time instants in order to find the average displacement trend along the height of the structure. By this way, the over- and under-estimation could be better interpreted, and more reliable results would be delivered.

The larger noise of the radar interferometer, that was mainly evident under natural vibrations (Figure 19), does not compromise the capability of the system to identify the natural frequency even if it can produce significant uncertainties in the frequency identification of higher vibration modes.

It is worth highlighting that the radar interferometer directly senses the displacements; thus, for damage assessment purposes, the TInRAR provides valuable data that cannot be obtained by accelerometer-based systems, such as the residual displacement produced by traumatic events such as earthquakes. Moreover, the data processing required to provide the displacement time-series is fast and light in terms of data manipulation. 

Figure 15 also points out a relevant property of the radar interferometer: the capability to provide information about the displacements at the top of the tower, i.e., where the maximum displacement occurs. In that position, in fact, it is often really hard or even impossible to install an accelerometer due to accessibility problems.

In conclusion, the comparison with the traditional approaches based on accelerometers showed some advantages in the use of TInRAR: the direct sensing of displacements that can provide valuable information for damage assessment purposes; good agreement with accelerometers guarantees the reliability of results; the capability to identify the natural frequencies of the structure to be used for training finite element models for structural simulations; the ability to remotely sense which prevents unsafe conditions for operators, as well as avoids the invasive installation of equipment over the structure. On the other side, it is also worth noting the disadvantages arising from the comparison with traditional accelerometers: TInRAR is only able to measure along the line of sight, thus complicating the geometric design and execution of the survey and increasing the cost in cases of complex motions of the structure; the higher noise makes frequency identification of higher vibration modes difficult; the principles of TInRAR technology do not allow the identification of specific points because it is a surface-based method that provides an average response coming from the sensed area.

## 7. Conclusions

The experimentation carried out on the Saint Prospero tower allowed to identify the modal parameters of the structure and the displacements induced by playing the major bell, weighing 2.4 tons, with 360-degree oscillations. The vibrations were measured by means of a terrestrial radar interferometer as well as by two kinds of accelerometers. The redundant monitoring system allowed to analyze, cross-check, and validate the performance of the TInRAR technology under both stressed and natural conditions. The maximum displacement due to the bell forcing was about ±5 mm.

The capability of the TInRAR technology to remotely observe a structure and provide information on almost any point of interest, even if it is inaccessible to operators due to the presence of unsafe conditions or if it is not suitable for the installation of accelerometers, represents a key point for the exploitation of such technology in structural health monitoring. In addition, the quick setup of the instrument, the direct measurements of displacements along with the quite rapid data processing make the radar interferometer suitable for emergencies. On the other hand, the limitations of detecting one-dimensional displacements only are fully addressed by the integration with accelerometers. A monitoring system that combines terrestrial radar interferometer with some accelerometers, indeed, would be able to achieve comprehensive results.

## Figures and Tables

**Table 1:** IBIS-FS radar interferometer technical specifications [28].

Parameter	Technical Specifications
Displacement accuracy	0.01 mm/0.1 mm (depending on range)
Operating range	Up to 1000 m
Range resolution *	0.5 m
Acquisition frequency	Up to 200 Hz
Power supply	110/220 Vac or 12 Vdc (Battery)
Battery autonomy	4 h
Weight	32 kg (full configuration with tripod)
Operating temperature	−20 °C to +55 °C

* Range resolution depends on the frequency bandwidth authorized by local radio regulation. As an example, in the USA and Europe, the bandwidth is limited to 200 MHz and the range resolution is 0.75 m.
